# Polymorphisms in the receptor for advanced glycation end-products (RAGE) gene and circulating RAGE levels as a susceptibility factor for non-alcoholic steatohepatitis (NASH)

**DOI:** 10.1371/journal.pone.0199294

**Published:** 2018-06-21

**Authors:** Rohini Mehta, Gladys Shaw, Peter Masschelin, Sean Felix, Munkzhul Otgonsuren, Ancha Baranova, Zachary Goodman, Zobair Younossi

**Affiliations:** 1 Betty and Guy Beatty Center for Integrated Research, Inova Fairfax Medical Campus, Falls Church, Virginia, United States of America; 2 Center for the Study of Chronic Metabolic Diseases, George Mason University, Fairfax, Virginia, United States of America; 3 Center for Liver Disease, Department of Medicine, Inova Fairfax Hospital, Falls Church, Virginia, United States of America; University of Miami School of Medicine, UNITED STATES

## Abstract

Non-alcoholic fatty liver disease (NAFLD) is a hepatic manifestation of metabolic syndrome and major cause of chronic liver disease in developed countries. Its prevalence is increasing in parallel with the prevalence of obesity and other components of the metabolic syndrome. As the liver is central to the clearance and catabolism of circulating advanced glycosylation end-products (AGEs), AGEs and their cognate receptors—RAGE (receptor for AGEs) system might be involved in NAFLD in obese patients. To examine this, we investigated four common polymorphisms of RAGE gene: 1704G/T (rs184003), G82S (rs2070600), -374T/A (rs1800624) and −429T/C (rs1800625) in 340 obese patients with metabolic syndrome. and protein levels of AGE and RAGE. This is the first study to describe association of 4 common polymorphisms with non-alcoholic steatohepatitis (NASH) as well as to examine protein levels of RAGE and AGE. Univariate analysis showed patients carrying the rs1800624 heterozygote genotype (AT) exhibited 2.36-fold increased risk of NASH (odds ratio (OR) = 2.36; 95% confidence interval (95% CI): 1.35–4.19) after adjusting for confounders. The minor allele -374 A has been shown to suppress the expression of RAGE protein. The protein levels of esRAGE, total sRAGE and AGE protein levels did not correlate with each other in obese patients with no liver disease, indicative of RAGE signaling playing an independent role in liver injury. In obese patients with non-NASH NAFLD and NASH respectively, esRAGE protein showed strong positive correlation with total sRAGE protein. Further, haplotype analysis of the 4 SNPs, indicated that haplotype G-A-T-G was significantly associated with 2-fold increased risk for NASH (OR = 2.08; 95% CI: 1.21–3.5; P = 0.006) after adjusting for confounders. In conclusion, the presented data indicate that the G-A-T-G haplotype containing minor allele at position −374 A and major allele at position −429T, 1704G, and G82S G could be regarded as a marker for NASH.

## Introduction

Non-alcoholic fatty liver disease (NAFLD) is a major cause of chronic liver disease in developed countries[1],[2]. NAFLD is considered to be the hepatic manifestation of metabolic syndrome [3]. Its prevalence is increasing in parallel with the prevalence of obesity and other components of the metabolic syndrome [2]. NAFLD includes a range of conditions, from simple steatosis characterized by fat accumulation in more than 5% of the hepatocytes [4] to non-alcoholic steatohepatitis (NASH) characterized by steatosis along with inflammation and/or ballooning degeneration, Mallory-denk bodies with/without pericellular fibrosis [5], [4]. NAFLD affects 25–30% of the general population, 2–5% have the subset of NASH and 1–2% of all NASH patients are at risk for progressing to cirrhosis [1], [6]. The progression of NASH to cirrhosis has been estimated to be 28 years[7]. However, the reasons for progression of NAFLD to NASH and cirrhosis are as yet unclear. NAFLD alone, is projected to be the leading indication for liver transplant within a decade[1], [8]. The major risk factors for NAFLD are the same as the components of the metabolic syndrome: central obesity, type 2 diabetes mellitus, dyslipidaemia and insulin resistance [1].

Advanced glycation end products (AGEs) are a complex and heterogeneous group of tissue bound and circulating glycol-oxidated proteins. The liver is central to the clearance and catabolism of circulating advanced glycosylation end-products (AGEs) [9]. Thus, AGEs and their cognate receptors—RAGE (receptor for AGEs) system might be involved in NAFLD in obese patients. The activation of RAGE has been shown to activate oxidative stress and subsequently evoke inflammatory responses as well as upregulate RAGE gene expression [10], [2], [7], [11].

RAGE is a receptor that can bind to multiple ligands (multi-ligand receptor). Under normal physiological conditions, RAGE expression is very low, with the lungs being an exception. However in the presence of metabolic conditions such as hyperlipidemia and insulin resistance, higher levels of RAGE receptor can be detected in tissues [12], [13], [14], [15]. Interestingly, RAGE receptor has several common functional variants. The full length RAGE receptor is membrane bound (mRAGE) [9]. On the other hand, soluble RAGE receptor (sRAGE) lacks the transmembrane and cytosolic domains. sRAGE can be generated as a result of alternative splicing of the encoding DNA (endogenous secretory isoform (esRAGE)) or proteolytic cleavage of the full-length mRAGE [16],[15],[8]. While, the truncated sRAGE variants cannot activate downstream signal transduction upon ligand binding, they can compete with mRAGE for the ligand and thus help in dampening the signaling. Further, as AGEs promote RAGE expression in positive feedback loop, the circulating sRAGE concentration can serve as a indicator of RAGE gene expression within tissues, thus allow detection of expression changes within tissues [9].

The gene for RAGE (*AGER*) is located on chromosome 6p21.3 in the MHC locus and consists of of a 1.7-kb 5′ flanking region, 10 introns, 11 exons and a 3`UTR [11], [17] [18]. To date, numerous genetic variants have been identified in the RAGE gene, the majority of which are single-nucleotide polymorphisms.

In this study, we investigated four common polymorphisms of RAGE gene: 1704G/T (rs184003), G82S (rs2070600), -374T/A (rs1800624) and −429T/C (rs1800625) in 340 obese patients with metabolic syndrome. We performed a retrospective study to determine the relationship between genetic polymorphisms in the RAGE gene and severity of NAFLD; potential multilocus interactions that affect the severity of NAFLD; the association between RAGE polymorphisms and RAGE levels; and the association between RAGE levels and AGE.

## 2. Materials and methods

### 2.1. Sample collection

This study has been approved by Internal Review Board of Inova Fairfax Hospital (Federal Assurance FWA00000573). Sample collection and storage was approved by Inova IRB #05.047. Written informed consent for sample collection and use of samples for research at the Betty and Guy Beatty Center for Integrated Research, Inova Fairfax Medical Campus facility was obtained from all patients included in this study. Samples used for this protocol had been previously collected and stored in -80 degree freezer. Fasting whole blood and serum samples processed from 340 patients (BMI > 35) undergoing weight loss surgery, were used for this study. In each sample, serum was separated and processed by standard procedure, then immediately flash frozen in liquid nitrogen and added to the repository of specimens stored at −80°C until use. The samples were de-identified in compliance with HIPAA regulations. A liver biopsy was performed at the time of surgery. Clinical and laboratory variables from the time of surgery were extracted from medical records and available for the study.

### 2.2 Diagnosis of NAFLD

All liver biopsies were read by same hepatopathologist. Other causes of chronic liver disease were excluded by negative serology for hepatitis B and C, no reported history of toxic exposure and excessive alcohol consumption (> 10 gram/day in women and >20 gram/day in men). Ethnicity was recorded as self-reported. Histological features such as portal inflammation, lymphoplasmacytic lobular inflammation, polymorphonuclear lobular inflammation, Kupffer cell hypertrophy, apoptotic bodies, focal parenchymal necrosis, glycogen nuclei, hepatocellular ballooning, and Mallory-Denk bodies were evaluated in the H & E sections. The extent of steatosis was graded based on an estimate of the percentage of tissue occupied by fat vacuoles as follows: 0 = none, 1 = <5%, 2 = 6–33%, 3 = 34–66%, 4 = >66%. NASH was defined as steatosis, lobular inflammation, and ballooning degeneration with or without Mallory Denk bodies, and with or without fibrosis. The extent of various immune cell infiltrations such as lymphoplasmacytic cells, polymorphonuclear cells and Kupffer cell hypertrophy was assessed by hematoxylin-eosin (H&E) staining. For each category, scores were assigned based on the following system: 0 = none, 1 = few, 2 = moderate, 3 = many. The extent of hepatic inflammation was determined based on the sum of the above individual scores with a score of ≥3 being considered as advanced hepatic inflammation and score of <3 being considered as mild/no hepatic inflammation. Severity of pericellular and portal fibrosis was determined by Masson trichrome staining of the biopsy, respectively. The scoring was as follows: 0 = no fibrosis, 1 = mild fibrosis, 2 = moderate fibrosis, 3 = marked fibrosis. Severity of total hepatic fibrosis was determined based on sum of the individual scores (pericellular and portal fibrosis) with score of≥3 being considered as advanced hepatic fibrosis and score of <3 being considered as mild/no hepatic fibrosis. Patients with hepatic steatosis or NASH were considered to have NAFLD.

### 2.3. Genomic DNA extraction

Total DNA was extracted from whole blood using QIAamp® kits in accordance with manufacturer’s instructions (Qiagen, USA). DNA was then quantified and quality assessed by spectrophotometer (GeneQuant 1300, General Electric). Additionally, to assess the integrity of extracted DNA, agarose gel electrophoresis was carried out. The gel was inspected for evidence of poor DNA quality visible as degradation/smearing. Each DNA sample was diluted to a final concentration of 10ng/uL prior to the genotyping assays.

### 2.4. RAGE variants genotyping

Genotyping for each subject was performed using a TaqMan SNP assays (Applied Biosystems, CA, USA). Genotyping of RAGE SNPs rs1800625 (C_8848033_1), rs1800624 (C_3293837_1), rs2070600 (C_15867521_20), rs184003 (C_2412456_10) was carried out on CFX96 PCR instrument (Biorad, USA) according to the manufacturer’s protocol (50°C for 2 min, 95°C for 10 min, and then 40 cycles of 95°C for 15 s and 60°C for 1.5 min). After PCR amplification, a post-PCR plate reads were carried out to generate allelic discrimination plot.

### 2.5. ELISA for AGE and RAGE proteins

Previously collected and frozen serum samples were used to determine levels of the circulating AGE and RAGE proteins by 96-well sandwich ELISA kits. Measurements for glycated protein levels in serum samples was performed using OxiSelect™ Advanced Glycation End Product (AGE) Competitive ELISA Kit (Cell Biolabs Inc, USA). To measure the concentration of human total sRAGE in serum, Quantikine Human RAGE immunoassay (RD Systems, Wiesbaden, Germany) was used. The ELISA is designed to measure extracellular domain of human RAGE with a sensitivity if 16.14 pg/mL. The concentration of endogenous secreted RAGE in serum was measured using B-Bridge esRAGE ELISA Kit (for esRAGE assay, B-Bridge International, Sunnyvale, US). The ELISA is designed to measure the unique C terminal of secreted human RAGE with a sensitivity of 25 pg/ml. In these ELISA assays, undiluted serum was used and measurements performed following the manufacturer's instructions. The results were expressed in pg/mL.

### 2.6. Statistical methods

Statistical analysis was performed using the SAS software package (SAS V9.3) for descriptive analysis and R (V3.3.3) for haplotype and linkage disequilibrium analysis. Continuous variables were expressed as the mean ± SD. Categorical variables were presented as frequencies. Group differences were analyzed by the Mann-Whitney U test, and the Chi-square test. For genotypic and allelic frequencies, the Hardy-Weinberg equilibrium was applied using SNPStats online tools. Univariate analysis and multiple logistic regression analysis were performed for haplogroup assessment of RAGE variants and RAGE protein levels in the subjects. A value of <0.05 was considered statistically significant for all analyses.

## Results

The clinical, demographic and biochemical characteristics of study subjects (N = 340; BMI = 48.1±9.08, Age (yrs) = 44±11.3, 30.3% NASH, 42.1% non-NASH NAFLD, 27.6% normal liver histology, 29.1% pericellular liver fibrosis) are summarized in **Table 1**. Among patients with NASH, 91.3% had pericellular fibrosis. Serum aminotransferases (ALT,AST), fasting serum glucose, serum triglycerides were higher in patients with histologic NASH when compared to patients with non-NASH NAFLD and normal liver histology (*p* < 0.001). In contrast, fasting serum HDL and platelets were lower in NASH patients compared to those with non-NASH NAFLD (*p* < 0.001) (**Table 2***)*.

**Table 1:** Demographic and clinical data of the patient cohorts profiled for expression of RAGE proteins and polymorphisms.

Demographic and Clinical Data	Mean ± SD (N = 340)
BMI	48.1±9.08
Age (yrs)	44±11.3
Non-NASH NAFLD	42.1% (143)
NASH	30.3% (103)
Normal liver histology/No liver disease	27.6% (93)
Pericellular liver fibrosis	29.1% (99)
ALT (U/L)	34.59±25.92
AST (U/L)	26.37±19.35
Glucose (mg/dL)	108.88±36.76
Triglycerides (mg/dL)	157.9±93.4
Total cholesterol (mg/dL)	187.6±39.3

BMI: Body Mass Index; AGE: Advanced Glycation End Products; esRAGE: Endogenous Receptor for Advanced Glycation Products; sRAGE: Soluble Receptor for Advanced Glycation; AST: Aspartate Aminotransferase; ALT: Alanine Aminotransferase; HDL: High Density Lipoproteins

**Table 2:** Characteristics of clinical and demographic data for the cohorts (Mean±SD [N]); p<0.001.

Clinical Data	No liver Disease	Non-NASH NAFLD	NASH
BMI	46.8±7.98	47.8±8.5	49.55±10.59
AGE (ug/mL)	10.37±4.68	9.32±4.74	10.14±5.31
esRAGE (ng/mL)	0.22±0.11	0.21±0.1	0.2±0.07
Total sRAGE (pg/mL)	894.8±579.8	1061.2±624.8	1096.13±458.98
ALT (U/L)*	22.2 ±9.7 [90]	31.6±17.2 [104]	49.5±37.1 [103]
AST (U/L)*	19.4±5.54 [90]	23.5±11.23 [104]	36.4±29.54 [103]
Glucose (mg/dL)*	94.16±21.6 [87]	110.5±35.6 [94]	120.2±43.3 [99]
Triglycerides (mg/dL)*	125.8±58.1 [83]	160.3±94.5 [92]	182.1±116.4 [91]
Total cholesterol (mg/dL)*	175.8±33.8 [83]	193.2±35.8 [93]	190.1±41.7 [93]
Platelets (x 10^3^/uL)*	303±69.3 [91]	291.0±65.1 [103]	268.7±66.6 [101]
HDL (mg/dL)*	50.4±12.3 [78]	48.6±12.9 [79]	42.7±10.9 [82]

BMI: Body Mass Index; AGE: Advanced Glycation End Products; esRAGE: Endogenous Receptor for Advanced Glycation Products; sRAGE: Soluble Receptor for Advanced Glycation; AST: Aspartate Aminotransferase; ALT: Alanine Aminotransferase; HDL: High Density Lipoproteins

* p value less than 0.005.

### Association of RAGE protein levels

Mann-Whitney analysis of AGE, total sRAGE, esRAGE protein levels in patients did not reveal any significant differences. Analysis of protein levels in patients with mild (≤0–2) and advanced steatosis (≥3–4) did not show any differences (**S1 Table**). The mean expression of total sRAGE was 918.21 pg/mL, esRAGE was 0.19 ng/mL and AGE was 9.84±1.38 (ug/mL).

### Spearman correlation of AGE with total sRAGE and esRAGE protein levels

In non-NASH NAFLD patients, esRAGE protein (ng/mL) positively and strongly correlated with total sRAGE (pg/mL) (r = 0.54;p<0.001) but not with AGE (ug/mL) (**Table 3**). Notably, AGE protein (ug/mL) was negatively correlated with total sRAGE protein (pg/mL) (r = -0.33;p = 0.0007) (**Table 3**). In NASH patients, esRAGE protein (ng/mL) positively and strongly correlated with total sRAGE (pg/mL) (r = 0.5;p<0.001) and AGE (ug/mL) (r = 0.36;p = 0.003), respectively (**Table 3**). In patients with no liver disease, no correlations were seen between AGE, total sRAGE and esRAGE levels indicative of absence of activated RAGE signaling (**Table 3**).

**Table 3:** Spearman correlations among AGE ligand, total sRAGE and esRAGE protein levels in the patient population. p<0.05 considered significant.

**AGE vs esRAGE**	**N**	**Rho**	**p**
NASH	59	0.3688	0.003
No liver disease	67	0.16	0.18
Non-NASH NAFLD	99	-0.031	0.76
**AGE vs total sRAGE**	**N**	**Rho**	**P**
NASH	59	0.0618	0.64
No liver disease	67	-0.1349	0.27
Non-NASH NAFLD	99	-0.3321	0.0007
**esRAGE vs total sRAGE**	**N**	**Rho**	**P**
NASH	59	0.5166	0.00002
No liver disease	67	-0.1349	0.27
Non-NASH NAFLD	99	0.5408	<0.000001

AGE: Advanced Glycation End Products; esRAGE: Endogenous Receptor for Advanced Glycation Products; sRAGE: Soluble Receptor for Advanced Glycation; NAFLD: Non-alcoholic Fatty Liver Disease; NASH: Non-alcoholic Steatohepatitis.

### Association of RAGE gene polymorphisms

The genotype distributions of the four common SNPs were examined in patient population. The observed genotype distributions of four examined SNPs in RAGE gene were consistent with the Hardy-Weinberg equilibrium in patients (P > 0.05). The genotype distributions and frequencies of four polymorphisms in RAGE gene between cohorts are presented in **Table 4**. Analysis of RAGE gene polymorphisms levels in patients with mild (≤0–2) and advanced steatosis (≥3–4) did not show any differences (**S1 Table**).Among these, rs1800624 (-374T/A) differed significantly between patients with NASH and normal liver histology (**Table 5**). Univariate analysis showed patients carrying the rs1800624 heterozygote genotype (AT) exhibited 2.36-fold increased risk of NASH (odds ratio (OR) = 2.36; 95% confidence interval (95% CI): 1.35–4.19). No association was seen between RAGE polymorphisms and RAGE protein levels (**S2–S5** Tables).

**Table 4:** Genotype distributions and frequencies of four polymorphisms in RAGE gene between cohorts. The major genotype for each SNP is highlighted in bold.

	rs184003(1704G/T)			rs1800624(-374T/A)			rs1800625(-429T/C)			rs2070600(G82S)		
	GG	GT	TT	TT	TA	AA	TT	TC	CC	GG	GA	AA
No liver Disease (94)	**75**(0.80)	18(0.19)	1(0.01)	**68**(0.72)	23(0.24)	3(0.03)	**69**(0.73)	21(0.22)	4(0.04)	**85**(0.90)	9(0.10)	0
Non-NASH NAFLD (143)	**116**(0.81)	24(0.17)	3(0.02)	**90**(0.63)	46(0.32)	7(0.05)	**99**(0.69)	39(0.27)	5(0.03)	**131**(0.92)	12(0.08)	0
NASH (103)	**90**(0.87)	12(0.12)	1(0.01)	**54**(0.52)	43(0.42)	6(0.06)	**70**(0.68)	30(0.29)	3(0.03)	**95**(0.02)	9(0.08)	0

**Table 5:** Chi-square analysis for associations between allele frequency at each SNP and different cohorts. Values are given as N (%). p≤ 0.05 considered significant.

SNP	Genotype	No liver Disease	NAFLD	NASH
rs2070600	GA	9 (9.57)	12 (8.39)	8 (7.77)
	GG	85 (90.43)	131 (91.61)	95 (92.23)
rs184003	GG	75 (79.79)	116 (81.12)	90 (87.38)
	GT	18 (19.15)	24 (16.78)	12 (11.65)
	TT	1 (1.06)	3 (2.1)	1 (0.97)
rs1800624†	AA	3 (3.19)	7 (4.9)	6 (5.83)
	AT	23 (24.47)	46 (32.17)	43 (41.75)
	TT	68 (72.34)	90 (62.94)	54 (52.43)
rs1800625	CC	4 (4.26)	5 (3.5)	3 (2.91)
	CT	21 (22.34)	39 (27.27)	30 (29.13)
	TT	69 (73.4)	99 (69.23)	70 (67.96)

† significant between No liver Disease and NASH

### Association of RAGE haplotypes with non-NASH NAFLD and NASH

The haplotypes derived from four examined polymorphisms in RAGE gene, are summarized in **Table 6**. The frequencies of haplotypes G-A-T-G (alleles in order rs184003, rs1800624, rs1800625, rs2070600) (Simulated P = 0.009) was significantly higher in NASH patients than in patients normal liver histology. Accordingly, univariate analysis compared with the most common haplotype G-T-T-G, indicated that haplotype G-A-T-G was significantly associated with 2-fold increased risk for NASH (OR = 2.08; 95% CI: 1.21–3.5; P = 0.006). Multivariate logistical analysis after adjusting for BMI, AGE, gender, showed a significant relationship between haplotype and risk for NASH (**Table 7**). G-A-T-G haplotype exhibited 2.14 increased risk of NASH (odds ratio (OR) = 2.14; 95% confidence interval (95% CI:1.09–4.21). However, no significant relationship was seen when AST and ALT where included in this model (**Table 8**).

**Table 6:** Univariate analysis for RAGE gene haplotypes, showing association with NASH in obese patients.

NASH (N = 103) vs No Liver Disease (N = 94)				
	OR	95% CI		P value
**G**-**T**-**T**-**G**	Reference			
T-**T**-**T**-**G**	0.65	0.28	1.49	0.14
**G**-**T**-**T**-A	0.92	0.32	2.58	0.65
**G**-**T**-C-G	1.30	0.73	2.33	0.57
**G**-A-**T-G**	**2.08**	**1.21**	**3.58**	**0.006**

Haplotype Analyses for outcome: (alleles in order rs184003, rs1800624, rs1800625, rs2070600). The major allele in each haplotype is highlighted in bold. OR: Odds ratio; CI: Confidence interval

**Table 7:** Multivariate analysis for RAGE gene haplotypes, showing association with NASH in obese patients after adjusting for BMI, Age, Gender.

Table				
Table	Table	95% CI		P value
Table	Table	1.01	1.07	0.015
Table	Table	1.68	6.95	<0.001
Table	Table	1.00	1.08	0.027
Table	Table	0.43	3.84	0.647
Table	Table	0.29	1.76	0.463
Table	Table	0.19	1.87	0.370
Table	Table	0.56	2.43	0.679
Table	Table	1.09	4.21	0.028

Haplotype Analyses for outcome: (alleles in order rs184003, rs1800624, rs1800625, rs2070600). The major allele in each haplotype is highlighted in bold. OR: Odds ratio; CI: Confidence interval

**Table 8:** Multivariate analysis for RAGE gene haplotypes, showing association with NASH in obese patients after adjusting for BMI, Age, Gender, AST and ALT.

NASH (N = 103) vs No Liver Disease (N = 94)				
	OR	95% CI		P value
AGE	1.04	1.00	1.08	0.04
Gender	1.37	0.58	3.25	0.47
BMI	1.05	1.01	1.09	0.021
ALT	1.05	1.01	1.10	0.024
AST	1.09	1.01	1.17	0.028
**G-T-T-G**	0.58	0.17	1.99	0.385
T-**T**-**T**-**G**	0.59	0.20	1.75	0.339
**G**-**T**-**T**-A	1.23	0.31	4.93	0.765
**G**-**T**-C-G	1.00	0.42	2.37	0.995
**G**-A-**T-G**	1.43	0.64	3.19	0.384

Haplotype Analyses for outcome: (alleles in order rs184003, rs1800624, rs1800625, rs2070600). The major allele in each haplotype is highlighted in bold. OR: Odds ratio; CI: Confidence interval

### Linkage disequilibrium

**Fig 1:**
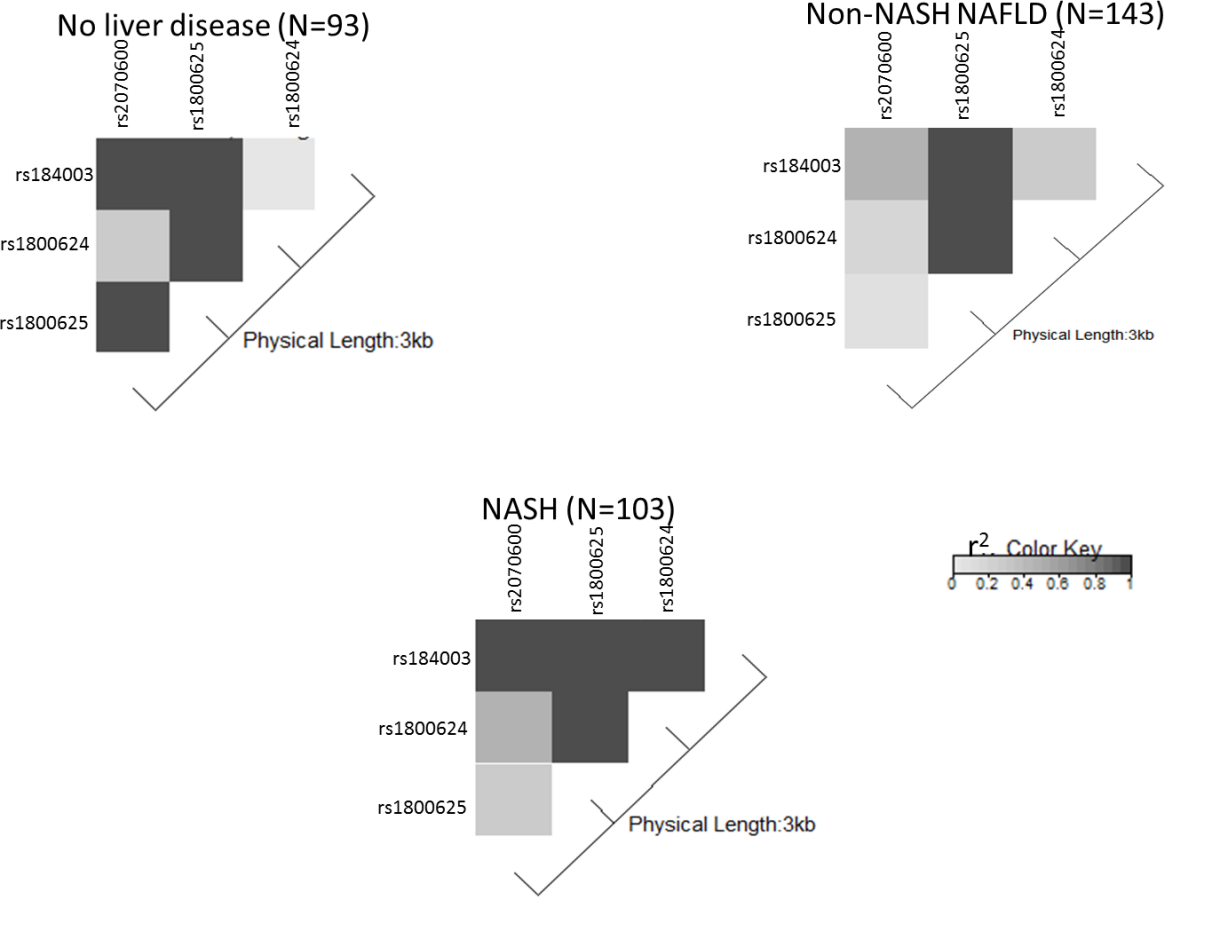
The intensity of the grey color in each pairwise SNP comparison is a measure of the strength of LD as measured by r^2^.Dark grey boxes indicate a r^2^ of 1 (100% linkage), and the gradation of color to white indicates progressively smaller r^2^, values indicative of weaker LD. Notes: Columns 1 to 3 are: rs2070600, rs1800625 and rs1800624; Rows 1 to 3 are: rs184003, rs1800624 and rs1800625.

Besides genotype analysis, we performed linkage disequilibrium to obtain information about correlation between SNPs. The pattern and extent of linkage disequilibrium (LD) at each genomic region differs (**Fig 1**). LD was measured using the statistic r^2^ and was plotted to illustrate the intensity of LD along the length of the gene spanned by our markers. The amount and pattern of LD varied between our cohorts. In the 3kb RAGE/AGER gene region, we observed the highest LD between SNPs rs184003 and rs2070600; rs184003 and rs1800625; rs1800625 and rs2070600; rs1800624 and rs1800625 (r^2^ = 1) in the No liver disease group (**Fig 1**). However, in non-NASH NAFLD, a weaker association was observed between SNPs rs184003 and rs2070600 (r^2^ = 0.6); rs1800625 and rs2070600 (r^2^ = 0.2); while a stronger association was seen between rs184003 and rs1800625 (r^2^ = 1), respectively. In NASH group, strong linkage disequilibrium was seen between rs184003 and the remaining three SNPs (r^2^ = 1) (**Fig 1**). rs1800624 was highly linked with rs1800625 in all the groups (**Fig 1**).

## Discussion

RAGE (Receptor for AGE) is a multiligand member of the immunoglobulin superfamily of cell surface molecules [19]. It was first described as a specific receptor for Advanced Glycation Endproducts (AGEs) [20]. Ligation of RAGE by one of its multitude ligand is reported to upregulate the expression of the receptor on a variety of cells such as monocytes, lymphocytes and endothelial cells [21]. The interaction also triggers a cascade of downstream pro-inflammatory signaling pathways that drive host response towards tissue destruction [19],[22],[23],[24]. Notably, studies have shown AGE-RAGE interaction to aggravate experimental hepatic fibrosis [24] and NAFLD [25]. esRAGE, a C-truncated splice variant of RAGE [26] has been proposed to influence RAGE signaling by functioning as a decoy receptor for RAGE ligands. Thus, circulating esRAGE levels can serve as 1) A marker of RAGE production within tissues [27]; 2) A response to attenuate RAGE induced tissue damage [28].

In our study, esRAGE, total sRAGE and AGE protein levels were not correlated with each other in obese patients with no liver disease (**Table 3**). This is supportive of RAGE signaling playing an independent role in liver injury. However, in patients with non-NASH NAFLD and NASH respectively, esRAGE protein showed strong positive correlation with total sRAGE protein (**Table 3**). This is supportive of role of esRAGE as a decoy for the cell surface RAGE protein and is indicative of dampening of RAGE- AGE axis by esRAGE in these patients [28].

The negative correlation between total sRAGE and AGE in non-NASH NAFLD but, lack of correlation in patients with NASH (**Table 2**) may be attributed to other RAGE ligands playing a role in RAGE pathway activation as NAFLD progresses to NASH. One of the limitations of the current study is only AGE ligand was measured. RAGE has several non-AGE ligands such as HMGB1, S1000 [29]. It’s plausible that in non-NASH NAFLD, initial AGE formation activates RAGE. Subsequently, immune cells become activated and release non- AGE RAGE ligands such as the S100 and HMGB1. To determine the extent and mechanism of RAGE pathway activation in NASH, additional ligands need to be measured. It would be interesting to explore if different mechanisms of RAGE axis activation are involved in non-NASH NAFLD development when compared to NASH.

In our study, we also determined the frequency of 4 common SNPs in RAGE gene. The G82S in the coding region was the first identified polymorphism in RAGE gene. The Gly82Ser polymorphism promotes glycosylation of RAGE with implications for the structure of the ligand binding region [12], [10]. Therefore this variant may affect RAGE function [30], [10], [12]. The other 2 polymorphisms were located in the promoter region -374 T/A and −429 T/C with marked influence on transcriptional activity of RAGE gene [31]. The minor allele -374 A suppresses the expression of RAGE. On the other hand, the minor allele -429 C polymorphism increases the expression of RAGE. 1704 G/T is an intron polymorphism.

While a number of recent studies have explored the RAGE polymorphisms in cancer [32], [33], [34], [35] and diabetes[36],[37], there are limited studies on the association of these common RAGE polymorphisms with NAFLD [38]. Only one study has explored RAGE gene polymorphisms in liver disease. Su et al., showed a correlation of another RAGE gene promoter polymorphisms −429T/C with the early stage of liver tumorigenesis and implicated its protective role in the progression of hepatocellular carcinoma (HCC) [38]. In our cohort, however the −429 T/C, 1704 G/T, and G82S polymorphisms did not show any association with NAFLD or NASH. The promoter polymorphism −374 T/A showed significant difference in frequency in NASH patients compared to those with no liver disease (**Table 5**).Univariate analysis showed patients carrying the rs1800624 heterozygote genotype (AT) exhibited 2.36-fold increased risk of NASH (odds ratio (OR) = 2.36; 95% confidence interval (95% CI): 1.35–4.19) after adjusting for confounders.

Single locus analysis does not give information on coinheritance of multiple loci on a chromosome. Haplotype approach, which looks a combination of alleles along the chromosome or segment of chromosome, on the other hand gives greater information. Haplotype analysis enables determining extent of association between loci as well as ability to predict occurrence of one SNP based on the occurrence of other SNP when linkage is high [39]. In the present study, we have for the first time explored the RAGE gene haplotype associated with NASH. The SNPs used to construct haplotypes all had minor allele frequencies of ≥0.05. Among the haplotypes, haplotype G-A-T-G (alleles in order 1704 G/T, -374 T/A, −429 T/C, G82S G/C) was significantly associated with 2-fold increased risk for NASH (OR = 2.08; 95% CI: 1.21–3.5; P = 0.006) after adjusting for confounders. The high association of haplotype could be either due to one or more of the alleles (1704 G/T, -374 T/A, −429 T/C, G82S G/C) in the haplogroup being a causal allele or due to an allele that is in the close vicinity of this 4kb region. Further, expansion of this region of *AGER* gene to include surrounding SNPs would help identify the true causal variant. Notably in our study, the finding that heterozygous genotype at -374T/A is associated with 2.36 increased risk of NASH and the joint occurrence of major alleles at 3 SNPS (−429 T, 1704 G, and G82S G) with the minor allele at -374 A contributes to the NASH susceptibility supports the presence of true causal variant within this haplotype. Similarity between the outcome of individual SNP analysis and haplotype analysis **(Tables 4–7)** suggests possible cis-interaction between the variants. The Cis-interaction between two variants might together contribute to the alteration in the expression levels of RAGE and/or its variants.

In order to determine if these association patterns are specific for a NASH, we calculated pairwise LD for the analyzed SNPs in the RAGE gene in each of the cohort. A strong LD (r^2^) is indicative of higher chance of non-random coinheritance of the loci. All the 4 SNPs in RAGE gene were within a 3kb region. The amount and pattern of LD between SNPs varied among our cohorts. In patients with NASH, strong LD was seen between 1704G/T and the remaining three SNPs (r^2^ = 1), while in non-NASH NAFLD, strongest LD was seen between 1704G/T and −429T/C (r^2^ = 1) (**Fig 1**). Interestingly, in no liver disease cohort, 1704G/T was in strong LD with all except -374T/A. In all the three cohorts, -374T/A was highly linked with -429T/C (**Fig 1**). Notably, G82S showed relatively low LD with marker associated with NASH (−374T/A) in our cohorts (**Fig 1**). Low LD of G82S might suggest that the inheritance of this SNP locus is independent of the common haplotypes in the RAGE gene. A similar finding between G82S and common haplotypes has been shown by Kanková, K et al., in patients with diabetic neuropathy [40].

Thus, we see that G-A-T-G haplotype containing minor allele at position −374 A and major allele at position −429T, 1704G, and G82S G could serve as an indicator for presence of NASH (**Tables 6 and 7**) in the obese population. This is the first study to describe association of 4 common polymorphisms with NASH as well as to examine protein levels of RAGE and AGE. Several limitations of this study merit special consideration. First, the retrospective design of this study has inherent drawbacks, and precludes causal inferences. Second, only four polymorphisms were examined in this study, and it is highly encouraged to incorporate other polymorphisms. Third, only the AGE ligand was measured. It would be interesting to explore if different ligands of RAGE are involved in non-NASH NAFLD development when compared to NASH in obese patients. Fourth, the study has a selection bias by focusing only on obese patients undergoing bariatric surgery, thus is restricted to RAGE-AGE signaling in obese patients with metabolic syndrome. The difficulty and ethical considerations in obtaining tissue samples and liver biopsies from the general population not undergoing any surgical procedures limits the selection of cohort in this study.

## Supporting information

S1 Table Distributions and frequencies of four polymorphisms and RAGE proteins based on severity of steatosis. BMI: Body Mass Index; AGE: Advanced Glycation End Products; esRAGE: Endogenous Receptor for Advanced Glycation Products; sRAGE: Soluble Receptor for Advanced Glycation; AST: Aspartate Aminotransferase; ALT: Alanine Aminotransferase; HDL: High Density Lipoproteins;* p value less than 0.005. The non-parametric p-value is calculated by the Kruskal-Wallis test for numerical covariates and Fisher's exact test for categorical covariates.
(DOCX)

S2 Table Association of RAGE polymorphism rs184003 with metabolic abnormalities, other polymorphisms and RAGE-AGE protein levels. BMI: Body Mass Index; AGE: Advanced Glycation End Products; esRAGE: Endogenous Receptor for Advanced Glycation Products; sRAGE: Soluble Receptor for Advanced Glycation; AST: Aspartate Aminotransferase; ALT: Alanine Aminotransferase; HDL: High Density Lipoproteins;* p value less than 0.005. *The non-parametric p-value is calculated by the Kruskal-Wallis test for numerical covariates and Fisher's exact test for categorical covariates.
(DOCX)

S3 Table Association of RAGE polymorphism rs1800624 with metabolic abnormalities, other polymorphisms and RAGE-AGE protein levels. BMI: Body Mass Index; AGE: Advanced Glycation End Products; esRAGE: Endogenous Receptor for Advanced Glycation Products; sRAGE: Soluble Receptor for Advanced Glycation; AST: Aspartate Aminotransferase; ALT: Alanine Aminotransferase; HDL: High Density Lipoproteins;* p value less than 0.005. *The non-parametric p-value is calculated by the Kruskal-Wallis test for numerical covariates and Fisher's exact test for categorical covariates.
(DOCX)

S4 Table Association of RAGE polymorphism rs1800625 with metabolic abnormalities, other polymorphisms and RAGE-AGE protein levels. (DOCX)

S5 Table Association of RAGE polymorphism rs2070600 with metabolic abnormalities, other polymorphisms and RAGE-AGE protein levels. BMI: Body Mass Index; AGE: Advanced Glycation End Products; esRAGE: Endogenous Receptor for Advanced Glycation Products; sRAGE: Soluble Receptor for Advanced Glycation; AST: Aspartate Aminotransferase; ALT: Alanine Aminotransferase; HDL: High Density Lipoproteins;* p value less than 0.005. * The non-parametric p-value is calculated by the Kruskal-Wallis test for numerical covariates and Fisher's exact test for categorical covariates.
(DOCX)
