# Supplementary material for: Polymorphisms in the receptor for advanced glycation end-products (RAGE) gene and circulating RAGE levels as a susceptibility factor for non-alcoholic steatohepatitis (NASH)
Source: PLoS One. 2018 Jun 21;13(6):e0199294. doi: 10.1371/journal.pone.0199294 (PMC6013208; doi:10.1371/journal.pone.0199294)
Supplement: S1 Table — BMI: Body Mass Index; AGE: Advanced Glycation End Products; esRAGE: Endogenous Receptor for Advanced Glycation Products; sRAGE: Soluble Receptor for Advanced Glycation; AST: Aspartate Aminotransferase; ALT: Alanine Aminotransferase; HDL: High Density Lipoproteins;* p value less than 0.005. The non-parametric p-value is calculated by the Kruskal-Wallis test for numerical covariates and Fisher's exact test for categorical covariates. (DOCX) [file pone.0199294.s001.docx]

**Table S1:** Distributions and frequencies of four polymorphisms and RAGE proteins based on severity of steatosis.

| **Clinical Data** | **Genotype** | **Mild Steatosis (≤0-2)** | **Advanced Steatosis(≥3)** | **P value** |
| --- | --- | --- | --- | --- |
| BMI | | 47.87±8.64 | 48.75±10.64 | 0.55 |
| rs184003 | GG | 220 (81.78) | 61 (85.92) | 0.78 |
|  | GT | 45 (16.73) | 9 (12.68) |  |
|  | TT | 4 (1.49) | 1 (1.41) |  |
| rs1800624 | AA | 13 (4.83) | 3 (4.23) | 0.16 |
|  | AT | 82 (30.48) | 30 (42.25) |  |
|  | TT | 174 (64.68) | 38 (53.52) |  |
| rs1800625 | CC | 9 (3.35) | 3 (4.23) | 0.34 |
|  | CT | 67 (24.91) | 23 (32.39) |  |
|  | TT | 193 (71.75) | 45 (63.38) |  |
| rs2070600 | GA | 25 (9.29) | 4 (5.63) |  |
|  | GG | 244 (90.71) | 67 (94.37) |  |
|  | AA | 0 | 0 |  |
| AGE (ug/mL) | | 9.94±4.78 | 9.44±5.35 | 0.368 |
| esRAGE (ng/mL) | | 0.21±0.1 | 0.18±0.07 | 0.26 |
| Total sRAGE(pg/mL) | | 997.79±597.25 | 1125.71±470.06 | 0.16 |

BMI: Body Mass Index; AGE: Advanced Glycation End Products; esRAGE: Endogenous Receptor for Advanced Glycation Products; sRAGE: Soluble Receptor for Advanced Glycation; AST: Aspartate Aminotransferase; ALT: Alanine Aminotransferase; HDL: High Density Lipoproteins;* p value less than 0.005.

The non-parametric p-value is calculated by the Kruskal-Wallis test for numerical covariates and Fisher's exact test for categorical covariates.
