# Supplementary material for: Polymorphisms in the receptor for advanced glycation end-products (RAGE) gene and circulating RAGE levels as a susceptibility factor for non-alcoholic steatohepatitis (NASH)
Source: PLoS One. 2018 Jun 21;13(6):e0199294. doi: 10.1371/journal.pone.0199294 (PMC6013208; doi:10.1371/journal.pone.0199294)
Supplement: S2 Table — BMI: Body Mass Index; AGE: Advanced Glycation End Products; esRAGE: Endogenous Receptor for Advanced Glycation Products; sRAGE: Soluble Receptor for Advanced Glycation; AST: Aspartate Aminotransferase; ALT: Alanine Aminotransferase; HDL: High Density Lipoproteins;* p value less than 0.005. *The non-parametric p-value is calculated by the Kruskal-Wallis test for numerical covariates and Fisher's exact test for categorical covariates. (DOCX) [file pone.0199294.s002.docx]

**Table S2:** Association of RAGE polymorphism rs184003 with metabolic abnormalities, other polymorphisms and RAGE-AGE protein levels.

| **rs184003 vs Clinical Data** | **Genotype** | **GG (N=281)** | **GT (N=54)** | **TT (N=5)** | **P value** |
| --- | --- | --- | --- | --- | --- |
| BMI | | 48.14±9.02 | 47.58±9.66 | 48.66±8.09 | 0.892 |
| rs1800624 | AA | 13 (4.63) | 3 (5.56) | 0 (0) | 0.842 |
|  | AT | 95 (33.81) | 15 (27.78) | 2 (40) |  |
|  | TT | 173 (61.57) | 36 (66.67) | 3 (60) |  |
| rs1800625 | CC | 11 (3.91) | 1 (1.85) | 0 (0) | 0.136 |
|  | CT | 78 (27.76) | 9 (16.67) | 3 (60) |  |
|  | TT | 192 (68.33) | 44 (81.48) | 2 (40) |  |
| rs2070600 | GA | 27 (9.61) | 2 (3.7) | 0 (0) | 0.36 |
|  | GG | 254 (90.39) | 52 (96.3) | 5 (100) |  |
| AGE (ug/mL) | | 9.83±4.85 | 10.38±5.09 | 6.07±2.77 | 0.140 |
| esRAGE (ng/mL) | | 0.21±0.1 | 0.23±0.09 | 0.2±0.1 | 0.26 |
| Total sRAGE(pg/mL) | | 977.71±599.86 | 1139.3±538.52 | 1092.96±449.54 | 0.14 |
| LDL (mg/dL) | | 106.97±35.04 | 114±38.93 | 147±28.28 | 0.13 |
| Total Cholesterol (mg/dL) | | 186.16±38.45 | 195.15±43.28 | 217±21.21 | 0.23 |
| Triglycerides (mg/dL) | | 158.75±95.45 | 152.25±84.37 | 136±48.08 | 0.64 |
| HDL (mg/dL) | | 47.32±13.34 | 47.42±10.81 | 43±2.83 | 0.82 |
| ALT (U/L) | | 33.88±22.95 | 38.64±38.87 | 27.5±13.92 | 0.79 |
| AST (U/L) | | 25.71±15.5 | 30.28±33.44 | 19.5±7.59 | 0.59 |
| Glucose (mg/dL) | | 109.17±37.5 | 107.59±34.21 | 121±33.81 | 0.42 |
| Ballooning advanced | | 92 (86.79) | 14 (13.21) | 0 (0) | 0.220 |
| Ballooning mild | | 189 (80.77) | 40 (17.09) | 5 (2.14) |  |

BMI: Body Mass Index; AGE: Advanced Glycation End Products; esRAGE: Endogenous Receptor for Advanced Glycation Products; sRAGE: Soluble Receptor for Advanced Glycation; AST: Aspartate Aminotransferase; ALT: Alanine Aminotransferase; HDL: High Density Lipoproteins;* p value less than 0.005.

*The non-parametric p-value is calculated by the Kruskal-Wallis test for numerical covariates and Fisher's exact test for categorical covariates.
