# Supplementary material for: Polymorphisms in the receptor for advanced glycation end-products (RAGE) gene and circulating RAGE levels as a susceptibility factor for non-alcoholic steatohepatitis (NASH)
Source: PLoS One. 2018 Jun 21;13(6):e0199294. doi: 10.1371/journal.pone.0199294 (PMC6013208; doi:10.1371/journal.pone.0199294)
Supplement: S3 Table — BMI: Body Mass Index; AGE: Advanced Glycation End Products; esRAGE: Endogenous Receptor for Advanced Glycation Products; sRAGE: Soluble Receptor for Advanced Glycation; AST: Aspartate Aminotransferase; ALT: Alanine Aminotransferase; HDL: High Density Lipoproteins;* p value less than 0.005. *The non-parametric p-value is calculated by the Kruskal-Wallis test for numerical covariates and Fisher's exact test for categorical covariates. (DOCX) [file pone.0199294.s003.docx]

**Table S3:** Association of RAGE polymorphism rs1800624 with metabolic abnormalities, other polymorphisms and RAGE-AGE protein levels.

| **rs1800624 vs Clinical Data** | **Genotype** | **AA (N=16)** | **AT (N=112)** | **TT (N=212)** | **P value** |
| --- | --- | --- | --- | --- | --- |
| BMI | | 47.03±8.42 | 48.54±10.13 | 47.88±8.55 | 0.960 |
| rs1800625 | CC | 0 (0) | 0 (0) | 12 (5.66) | **0.001*** |
|  | CT | 0 (0) | 28 (25) | 62 (29.25) |  |
|  | TT | 16 (100) | 84 (75) | 138 (65.09) |  |
| rs1800624 | GG | 13 (81.25) | 95 (84.82) | 173 (81.6) | 0.84 |
|  | GT | 3 (18.75) | 15 (13.39) | 36 (16.98) |  |
|  | TT | 0 (0) | 2 (1.79) | 3 (1.42) |  |
| rs2070600 | GA | 0 (0) | 3 (2.68) | 26 (12.26) | **0.005*** |
|  | GG | 16 (100) | 109 (97.32) | 186 (87.74) |  |
| AGE (ug/mL) | | 7.45±3.67 | 9.96±4.76 | 9.94±4.99 | 0.322 |
| esRAGE (ng/mL) | | 0.17±0.09 | 0.23±0.09 | 0.21±0.11 | 0.160 |
| Total sRAGE(pg/mL) | | 837.34±309.83 | 1146.9±617.78 | 966.01±581.59 | 0.127 |
| LDL (mg/dL) | | 109.2±49.27 | 109.2±37.06 | 107.88±33.8 | 0.777 |
| Total Cholesterol (mg/dL) | | 194.67±44.83 | 189.63±38.78 | 186.22±39.21 | 0.63 |
| Triglycerides (mg/dL) | | 143.73±62.84 | 164.98±82.15 | 154.63±101 | 0.157 |
| HDL (mg/dL) | | 49.2±11.82 | 46.13±12.1 | 47.83±13.46 | 0.50 |
| ALT (U/L) | | 39.81±35.73 | 36.6±26.67 | 33.04±24.81 | 0.07 |
| AST (U/L) | | 26.19±16.71 | 28.13±24.3 | 25.41±16.45 | 0.16 |
| Glucose (mg/dL) | | 107.19±32.51 | 107.29±34.81 | 110.22±38.41 | 0.80 |
| Ballooning advanced | | 6 (5.66) | 42 (39.62) | 58 (54.72) | 0.138 |
| Ballooning mild | | 10 (4.27) | 70 (29.91) | 154 (65.81) |  |

BMI: Body Mass Index; AGE: Advanced Glycation End Products; esRAGE: Endogenous Receptor for Advanced Glycation Products; sRAGE: Soluble Receptor for Advanced Glycation; AST: Aspartate Aminotransferase; ALT: Alanine Aminotransferase; HDL: High Density Lipoproteins;* p value less than 0.005.

*The non-parametric p-value is calculated by the Kruskal-Wallis test for numerical covariates and Fisher's exact test for categorical covariates.
