# Supplementary material for: Polymorphisms in the receptor for advanced glycation end-products (RAGE) gene and circulating RAGE levels as a susceptibility factor for non-alcoholic steatohepatitis (NASH)
Source: PLoS One. 2018 Jun 21;13(6):e0199294. doi: 10.1371/journal.pone.0199294 (PMC6013208; doi:10.1371/journal.pone.0199294)
Supplement: S4 Table — (DOCX) [file pone.0199294.s004.docx]

**Table S4:** Association of RAGE polymorphism rs1800625 with metabolic abnormalities, other polymorphisms and RAGE-AGE protein levels.

| **rs1800625 vs Clinical Data** | **Genotype** | **AA (N=16)** | **AT (N=112)** | **TT (N=212)** | **P value** |
| --- | --- | --- | --- | --- | --- |
| BMI | | 46.55±7.51 | 50.45±11.46 | 47.22±7.94 | 0.17 |
| rs184003 | GG | 11 (91.67) | 78 (86.67) | 192 (80.67) | 0.136 |
|  | GT | 1 (8.33) | 9 (10) | 44 (18.49) |  |
|  | TT | 0 (0) | 3 (3.33) | 2 (0.84) |  |
| rs1800624 | AA | 0 (0) | 0 (0) | 16 (6.72) | **0.001*** |
|  | AT | 0 (0) | 28 (31.11) | 84 (35.29) |  |
|  | TT | 12 (100) | 62 (68.89) | 138 (57.98) |  |
| rs2070600 | GA | 0 (0) | 10 (11.11) | 19 (7.98) | 0.51 |
|  | GG | 12 (100) | 80 (88.89) | 219 (92.02) |  |
| AGE (ug/mL) | | 11.57±7.78 | 9.93±5.15 | 9.74±4.66 | 0.912 |
| esRAGE (ng/mL) | | 0.18±0.1 | 0.2±0.08 | 0.22±0.11 | 0.718 |
| Total sRAGE(pg/mL) | | 1027.06±714.43 | 864.25±500.69 | 1051.61±602.48 | 0.291 |
| LDL (mg/dL) | | 107.09±28.64 | 110.12±36.28 | 107.95±36.15 | 0.79 |
| Total Cholesterol (mg/dL) | | 180.92±33.33 | 191.34±39.87 | 186.85±39.43 | 0.4 |
| Triglycerides (mg/dL) | | 156±76.24 | 159.99±73.25 | 156.75±100.93 | 0.157 |
| HDL (mg/dL) | | 41.36±16.94 | 48.48±16.35 | 47.24±11.21 | 0.50 |
| ALT (U/L) | | 24.25±7.65 | 35.69±22.19 | 34.66±27.86 | 0.24 |
| AST (U/L) | | 19.92±5.35 | 25.34±10.7 | 27.07±22.19 | 0.27 |
| Glucose (mg/dL) | | 121.25±24.03 | 110.16±36.48 | 108.01±37.57 | **0.04*** |
| Ballooning advanced | | 2 (1.89) | 34 (32.08) | 70 (66.04) | 0.20 |
| Ballooning mild | | 10 (4.27) | 56 (23.93) | 168 (71.79) |  |
