# Supplementary material for: Polymorphisms in the receptor for advanced glycation end-products (RAGE) gene and circulating RAGE levels as a susceptibility factor for non-alcoholic steatohepatitis (NASH)
Source: PLoS One. 2018 Jun 21;13(6):e0199294. doi: 10.1371/journal.pone.0199294 (PMC6013208; doi:10.1371/journal.pone.0199294)
Supplement: S5 Table — BMI: Body Mass Index; AGE: Advanced Glycation End Products; esRAGE: Endogenous Receptor for Advanced Glycation Products; sRAGE: Soluble Receptor for Advanced Glycation; AST: Aspartate Aminotransferase; ALT: Alanine Aminotransferase; HDL: High Density Lipoproteins;* p value less than 0.005. * The non-parametric p-value is calculated by the Kruskal-Wallis test for numerical covariates and Fisher's exact test for categorical covariates. (DOCX) [file pone.0199294.s005.docx]

**Table S5:** Association of RAGE polymorphism rs2070600 with metabolic abnormalities, other polymorphisms and RAGE-AGE protein levels.

| **rs2070600 vs Clinical Data** | **Genotype** | **GA (N=29)** | **GG (N=311)** | **P value** |
| --- | --- | --- | --- | --- |
| BMI | | 48.91±8.26 | 47.98±9.17 | 0.17 |
| rs184003 | GG | GG | 27 (93.1) | 0.36 |
|  | GT | GT | 2 (6.9) |  |
|  | TT | TT | 0 (0) |  |
| rs1800624 | AA | AA | 0 (0) | **0.005*** |
|  | AT | AT | 3 (10.34) |  |
|  | TT | TT | 26 (89.66) |  |
| rs1800625 | CC | CC | 0 (0) | 0.5 |
|  | CT | CT | 10 (34.48) |  |
|  | TT | TT | 19 (65.52) |  |
| AGE (ug/mL) | | 9.75±4.88 | 9.85±4.89 | 0.94 |
| esRAGE (ng/mL) | | 0.17±0.08 | 0.22±0.1 | 0.13 |
| Total sRAGE(pg/mL) | | 805.06±342.26 | 1029.11±600.62 | 0.28 |
| LDL (mg/dL) | | 101.8±34.85 | 108.97±35.9 | 0.52 |
| Total Cholesterol (mg/dL) | | 180±34.26 | 188.38±39.63 | 0.37 |
| Triglycerides (mg/dL) | | 142.77±36.87 | 158.73±96.4 | 0.8 |
| HDL (mg/dL) | | 47.3±8.84 | 47.31±13.18 | 0.87 |
| ALT (U/L) | | 29.96±16.67 | 34.96±26.68 | 0.49 |
| AST (U/L) | | 22.44±10.16 | 26.7±20.02 | 0.23 |
| Glucose (mg/dL) | | 109.54±40.14 | 109.03±36.64 | 0.92 |
| Ballooning advanced | | 5 (4.72) | 101 (95.28) | 0.09 |
| Ballooning mild | | 24 (10.26) | 210 (89.74) |  |

BMI: Body Mass Index; AGE: Advanced Glycation End Products; esRAGE: Endogenous Receptor for Advanced Glycation Products; sRAGE: Soluble Receptor for Advanced Glycation; AST: Aspartate Aminotransferase; ALT: Alanine Aminotransferase; HDL: High Density Lipoproteins;* p value less than 0.005.

* The non-parametric p-value is calculated by the Kruskal-Wallis test for numerical covariates and Fisher's exact test for categorical covariates.
